# Supplementary material for: Evaluating livestock farmers knowledge, beliefs, and management of arboviral diseases in Kenya: A multivariate fractional probit approach
Source: PLoS Negl Trop Dis. 2021 Sep 16;15(9):e0009786. doi: 10.1371/journal.pntd.0009786 (PMC8478187; doi:10.1371/journal.pntd.0009786)
Supplement: Attachment — Submitted filename: PNTD-D-21-00736_Review.docx [file pntd.0009786.s002.docx]

PLOS Neglected Tropical Diseases

Evaluating Knowledge, Beliefs, and Management of Arboviral Diseases in Kenya: A

Multivariate Fractional Probit Approach

--Manuscript Draft--

| Manuscript Number: | PNTD-D-21-00736 |
| --- | --- |
| Full Title: | Evaluating Knowledge, Beliefs, and Management of Arboviral Diseases in Kenya: A Multivariate Fractional Probit Approach |
| Short Title: | Knowledge, Beliefs, and Management of ADs in Kenya |
| Article Type: | Research Article |
| Keywords: | Arboviral Diseases, Beliefs, Knowledge, Management, Multivariate Fractional Probit Model |
| Abstract: | Globally, arthropod-borne virus (arbovirus) infections continue to pose substantial threats to public health and economic development, especially in developing countries. In Kenya, although arboviral diseases (ADs) are largely endemic, little is known about the factors influencing livestock farmers’ knowledge, beliefs, and management (KBM) of the three major ADs: Rift Valley fever (RVF), dengue fever and chikungunya fever. This study evaluates the drivers of KBM of ADs from a sample of 629 respondents selected using a three-stage sampling procedure in Kenya’s three hotspot counties of Baringo, Kwale, and Kilifi. A multivariate fractional probit (MVFP) model was used to assess the factors influencing the intensity of KBM. Only a quarter of the farmers had any knowledge of the ADs while a vast majority of them (88 percent) could not manage any of the three diseases. Access to information (experience and awareness), income, education, religion and distance to a health facility significantly influenced the intensity of farmers KBM of ADs in Kenya. Thus, initiatives geared towards improving access to information through massive awareness campaigns are necessary to mitigate behavioral barriers in ADs management among rural communities in Kenya. |
| Additional Information: |  |
| Question | Response |
| Financial Disclosure  Enter a financial disclosure statement that describes the sources of funding for the work included in this submission. Review the [submission guidelines](http://journals.plos.org/plosntds/s/submission-guidelines#loc-financial-disclosure-statement) for detailed requirements. View published research articles from [PLOS NTDs](http://journals.plos.org/plosntds/) for specific examples.  This statement is required for submission and will appear in the published article if the submission is accepted. Please make sure it is accurate. | This study has been supported by funding from the Charité -Universitätsmedizin Berlin, Grant number- JU2857/9-1. The funders had no role in study design, data collection and analysis, decision to publish, or preparation of the manuscript. |

| Unfunded studies Enter: The author(s) received no specific funding for this work. Funded studies Enter a statement with the following details: - Initials of the authors who received each award - Grant numbers awarded to each author - The full name of each funder - URL of each funder website - Did the sponsors or funders play any role in the study design, data collection and analysis, decision to publish, or preparation of the manuscript? - NO - Include this sentence at the end of your statement: The funders had no role in study design, data collection and analysis, decision to publish, or preparation of the manuscript. - YES - Specify the role(s) played. |  |
| --- | --- |
| * typeset |  |
| Competing Interests  Use the instructions below to enter a competing interest statement for this submission. On behalf of all authors, disclose any [competing interests](http://journals.plos.org/plosntds/s/competing-interests) that could be perceived to bias this work—acknowledging all financial support and any other relevant financial or nonfinancial competing interests.  This statement will appear in the published article if the submission is accepted. Please make sure it is accurate. View published research articles from [PLOS NTDs](http://journals.plos.org/plosntds/) for specific examples. | The authors have declared that no competing interests exist. |

| NO authors have competing interests Enter: The authors have declared that no competing interests exist. Authors with competing interests Enter competing interest details beginning with this statement: I have read the journal's policy and the authors of this manuscript have the following competing interests: [insert competing interests here] |  |
| --- | --- |
| * typeset  This statement is required for submission and will appear in the published article if the submission is accepted. Please make sure it is accurate and that any funding sources listed in your Funding Information later in the submission form are also declared in your Financial Disclosure statement. |  |
| Data Availability  Authors are required to make all data underlying the findings described fully available, without restriction, and from the time of publication. PLOS allows rare exceptions to address legal and ethical concerns. See the [PLOS Data Policy](http://journals.plos.org/plosntds/s/data-availability) and [FAQ](http://journals.plos.org/plosntds/s/data-availability#loc-faqs-for-data-policy) for detailed information. | Yes - all data are fully available without restriction |

| A Data Availability Statement describing where the data can be found is required at submission. Your answers to this question constitute the Data Availability Statement and will be published in the article, if accepted. |  |
| --- | --- |
| Important: Stating ‘data available on request from the author’ is not sufficient. If your data are only available upon request, select ‘No’ for the first question and explain your exceptional situation in the text box. |  |
| Do the authors confirm that all data underlying the findings described in their manuscript are fully available without restriction? |  |
| Describe where the data may be found in full sentences. If you are copying our sample text, replace any instances of XXX with the appropriate details.   - If the data are held or will be held in a public repository, include URLs, accession numbers or DOIs. If this information will only be available after acceptance, indicate this by ticking the box below. For example: All XXX files   are available from the XXX database (accession number(s) XXX, XXX.).   - If the data are all contained within the manuscript and/or Supporting Information files, enter the following: All relevant data are within the manuscript and its Supporting Information files. - If neither of these applies but you are able to provide details of access elsewhere, with or without limitations, please do so. For example:   Data cannot be shared publicly because | The data is held in a public repository: https://doi.org/10.5061/dryad.ngf1vhhtt |

| of [XXX]. Data are available from the XXX Institutional Data Access / Ethics Committee (contact via XXX) for researchers who meet the criteria for access to confidential data.  The data underlying the results presented in the study are available from (include the name of the third party and contact information or URL).  • This text is appropriate if the data are owned by a third party and authors do not have permission to share the data.  * typeset |  |
| --- | --- |
| Additional data availability information: | Tick here if the URLs/accession numbers/DOIs will be available only after acceptance of the manuscript for publication so that we can ensure their inclusion before publication. |

Manuscript	[Click here to access/download;Manuscript;Nyangau et al - 2021](https://www.editorialmanager.com/pntd/download.aspx?id=1013477&guid=fc3ec1e0-22d2-4a72-8e69-1346f1d3c93c&scheme=1)

[- PLOSNTD.docx](https://www.editorialmanager.com/pntd/download.aspx?id=1013477&guid=fc3ec1e0-22d2-4a72-8e69-1346f1d3c93c&scheme=1)

# Evaluating Knowledge, Beliefs, and Management of Arboviral Diseases in Kenya: A

**Multivariate Fractional Probit Approach**

Paul Nyamweya Nyangau *^1^, Jonathan Makau Nzuma^1^, Patrick Irungu^1^ and Menale Kassie^2^

1 Department of Agricultural Economics, University of Nairobi, Kenya
2 International Centre of Insect Physiology and Ecology (*icipe*)

**^*^** Corresponding author: pnyangau@icipe.org

# Abstract

Globally, arthropod-borne virus (arbovirus) infections continue to pose substantial threats to public health and economic development, especially in developing countries. In Kenya, although arboviral diseases (ADs) are largely endemic, little is known about the factors influencing livestock farmers’ knowledge, beliefs, and management (KBM) of the three major ADs: Rift

Valley fever (RVF), dengue fever and chikungunya fever. This study evaluates the drivers of KBM of ADs from a sample of 629 respondents selected using a three-stage sampling procedure in Kenya’s three hotspot counties of Baringo, Kwale, and Kilifi. A multivariate fractional probit (MVFP) model was used to assess the factors influencing the intensity of KBM. Only a quarter of the farmers had any knowledge of the ADs while a vast majority of them (88 percent) could not manage any of the three diseases. Access to information (experience and awareness), income, education, religion and distance to a health facility significantly influenced the intensity of

farmers KBM of ADs in Kenya. Thus, initiatives geared towards improving access to

information through massive awareness campaigns are necessary to mitigate behavioral barriers in ADs management among rural communities in Kenya.

**Keywords:** Arboviral Diseases, Beliefs, Knowledge, Management, Multivariate Fractional Probit Model

## **1. Introduction**

Arthropod-borne viruses (arboviruses), known to be transmitted between vertebrate hosts and arthropod vectors, constitute a significant concern for global public health (Girard *et al*., 2020). Historically, arboviruses have caused notable diseases such as chikungunya fever, dengue fever, Rift Valley fever (RVF), yellow fever, and zika fever, leading to animal and human morbidity and mortality. Infections in humans and animals with clinical manifestations could range from subclinical to life-threatening conditions. For example, approximately 96 million symptomatic dengue cases and an estimated 40,000 dengue deaths are reported every year (WHO, 2020). The zoonotic effect of arboviral diseases (ADs) includes the decline in household income by reducing livestock stock and product sales and consumption, and the increase in household vulnerability in cases where livestock is used as a risk-coping mechanism (Birol *et al*., 2013).

In Kenya, multiple AD outbreaks have resulted in substantial economic losses and public health distress in the past three decades. These include the yellow fever outbreaks of 1992, 1995, and

2016 (Okello *et al*., 1993; Atoni *et al*., 2018; WHO, 2016); chikungunya fever in 2004 and 2016

(Sergon *et al*., 2008; Atoni *et al*., 2018); RVF incursions in 1997 and 2006 (Sergon *et al*., 2008; Munyua *et al*., 2010), and dengue fever outbreaks of 2011-2014 and 2017 (Atoni *et al*., 2018). These outbreaks resulted in widespread abortion and death of livestock and reduced milk, wool production, livestock growth, working days in humans, and draft animals (FAO, 2016). In rural communities where agriculture is the dominant livelihood source, the ADs can cause significant health and economic losses. For example, the 2007 RVF outbreak in Kenya contributed to economic losses estimated at US$32 million (Rich and Wanyoike, 2010).

The incidence of ADs is increasing, not just in East Africa but also in many regions of the world. This is due to several factors, including climate change, increased agricultural activity, and ecosystem changes (Caminade *et al*., 2019). Global warming, deforestation, and urbanization have led to a rapid expansion of the vectors’ habitats and have caused an enormous increase in vector-borne diseases worldwide (Marchi *et al*., 2018). Besides, the growing movement across regions of people and livestock has contributed to the broader distribution of the vectors that transmit emerging infectious diseases (Braack *et al*., 2018).

The effective management of the ADs depends on people’s perceptions of the disease, which in turn, are influenced by the availability of information for decision making as well as the level of knowledge and skills in disease management (Aerts *et al*., 2020; Alahdal *et al*., 2020). Previous studies reveal the limited awareness of ADs vectors, signs and symptoms among communities and livestock keepers in East Africa (Mangesho *et al*., 2017; Maurice *et al*., 2018; Owange *et al*., 2014; Shabani *et al*., 2015; Wensman *et al*., 2015). Other studies show poor management regarding ADs (Abdi *et al*., 2015; Higuera-Mendieta *et al*., 2016; Nguyen *et al*., 2019).

Evaluating community knowledge, beliefs, and management practices (KBM) of ADs is relevant for better policy guidance and investment in improving the affected communities’ health and economic status. The study on the KBM of ADs is also useful for setting a research agenda and developing targeted communication messages. Although, KBM studies have been undertaken previously in Eastern Africa on RVF ( Abdi *et al*., 2015; Affognon *et al*., 2017; Mangesho *et al*., 2017; Maurice *et al*., 2018; Owange *et al*., 2014; Shabani *et al*., 2015; Wensman *et al*., 2015), to the best of our knowledge, no study has examined the KBM of a portfolio of ADs (RVF,

Chikungunya fever, and Dengue fever) and their drivers in the region.

The failure to examine the KBM in a portfolio format has important implication in terms of accurate risk assessment with impact on the prevention and control of arbovirus infections (Dente *et al*., 2020). Even where KBM studies were undertaken for RVF, the studies used few respondents in one district. For example, Abdi *et al*., (2015) assessed KBM of RVF among 392 pastoralists living in Ijara district. Similarly, Owange *et al*., (2014) assessed risk factors of RVF among 31 key informants in Ijara district. This study assessed the KBM for three ADs in three hotspot counties in Kenya, namely, Baringo, Kilifi, and Kwale.

Our analysis contributes to the current limited empirical literature on KBM of ADs in the following ways. First, no KBM study has been conducted in the three ADs hotspot counties in the past. Second, our study employs a multivariate probit (MVP) analysis that considers the potential correlation between the KBM across different diseases to assess the socioeconomic and cultural factors that influence household health behavior. Finally, the study used the multivariate fractional probit (MVFP) model that considers the proportion of the correct answers provided by households for each outcome variable to estimate the intensity of KBM. We find low levels of knowledge and poor managerial skills of ADs that are largely driven by access to information and asset ownership.

The remainder of this paper is organized as follows. Section 2 presents the analytical framework of the study and discusses its empirical framework, the study area, the sampling procedure and measurement of the key variables used in the study. The empirical results are presented in section 3 while section 4 discusses them. Finally, section 5 concludes the paper and draws some policy recommendations.

**2. Methodology**

## ***2.1.Analytical Framework***

The Theory of Planned Behaviour (TPB) has been widely used in explaining the relationship between disease management and health-related outcomes (Ellis-Iversen *et al*., 2010; Mingolla *et al*., 2019; Vande Velde *et al*., 2015). The TPB is an extension of the Theory of Reasoned Action (TRA) that explains and predicts human behaviour. The TPB argues that decisions on certain behaviours result from a reasoned process (Sommer, 2011). Despite its wide-spread use, a common criticism of the TBP is that it does not account for other factors that might influence intention and motivation of individuals (Kan and Fabrigar, 2017). According to the TPB, three conceptually independent factors determine a person’s intention to manage diseases: attitude (A) towards the behaviour of interest (BI); subjective norms (SN); and perceived behavioural control (PBC), which can be presented as:

*BI=*𝑤_1_𝐴 + 𝑤_2_𝑆𝑁 + 𝑤_3_𝑃𝐵𝐶 ...................................................................................................... (1)

Where 𝑤_1_, 𝑤_2_ and 𝑤_3_ are the relative weights of attitudes, subjective norms, and PBC (Fishbein and Ajzen, 1977). The TPB posits that a person’s attitude (*A*) towards the behaviour of interest is based on readily accessible beliefs regarding the behaviour’s likely consequences (Ajzen, 2020):

*A*∝∑*b_i_e_i_* ………………………………………………………………………………………. (2)

Where *b* is the accessible belief for consequence *i* and *e* is the subjective evaluation of the outcome. On the other hand, subjective norms (SN) refer to the perceived social pressure to perform or not to perform the behaviour of interest (Fishbein and Ajzen, 1977). Following Ajzen, (2020), the SN are a function of an individual’s normative beliefs (*n*), and the significance

(*s*) to comply with the expectations (Equation 3);

SN∝∑𝑛*_i_*𝑠*_i_* …………………………………………………………………………………… (3) The PBC is a function of the composite score derived by summing the products of control belief strength (*c*) times perceived power (*p*) over all accessible control factors such as time, skills, money, and other resources expectations (Ajzen, 2020):

PBC∝∑𝑐*_i_*𝑝*_i_*…………………………………………………………………………………… (4)

We assume that the occurrence of one outcome may be conditional on the occurance of another outcome, with the correlation between them being either positive or negative (Khanna, 2001). In particular, a knowledgeable household might display positive beliefs or sound management practices towards a disease (Dhimal *et al*., 2014; Harapan *et al*., 2018). Knowing the disease signs and symptoms can allow timely recognition of the disease when it occurs. Further, households knowledgeable about a particular disease may adopt measures to prevent or quickly seek out either human or animal health services when there is an outbreak. Failure to capture such correlation and estimate the determinants of KBM separately using binary choice models could lead to biased and inefficient estimates (Kassie *et al*., 2015; Muriithi *et al*., 2018).

## ***2.2.Empirical model***

We employ a MVP model to operationalize equation 1 and account for the interdependence between the outcome variables (Chib and Greenberg, 1998; Song and Lee, 2005; Young *et al*., 2009; Kassie *et al*., 2015). Following Young *et al*., (2009), knowledge (*K*), belief (*B*) and management (*M*) of different diseases are a binary function of the decision maker’s

characteristics and can be modelled using the MVP regression as follows:

*K=β^k^_0_+β^k^_1_X_1_………+**K=1 if K>0, 0 otherwise*……………………………………… (5)

*B=β^b^_0_+β^b^_1_X_1_………+**B=1 if B>0, 0 otherwise* …………………………………….... (6)

*M=β^m^_0_ +β^m^_1_ X_1_………+β*_m_*^m^X*_m_*+ϵ^m^, M=1 if M>0, 0 otherwise* ……………………………...…… (7) where 𝛽 is the vector of parameters to be estimated, 𝑋 is a vector of decision maker’s characteristics (see for example, Affognon *et al*., 2017; Harapan *et al*., 2018; Mallhi *et al*., 2018), and 𝜖 is a vector of the error term. In the multivariate model, the error terms jointly follow a multivariate normal distribution (MVN) with zero conditional mean and variance normalized to unity for identification of the parameters, (𝜖~𝑀𝑁𝑉(0, Ω)), where Ω is the symmetric covariance matrix defined as:

…………………………..…………………………………………… (8)

where 𝜌 is the unobserved correlation of the KBM equations. A significant 𝜌 indicates interdependence between the error terms. A positive value of 𝜌 is considered “promotive” between the measured pair of equations, while a negative value of 𝜌 is “substitutive” (Ma *et al*.,

2018). The STATA command “mvprobit” was used to estimate the parameters 𝛽 and 𝜌.

The above model specification measures the determinants of the binary dependent variables (K, B, and M) with no distinction made between respondents that correctly answered one, two, three, or more knowledge-related questions. In other words, it ignores heterogeneity and/or knowledge intensity differences among the respondents. To correct this anomaly, the MVFP model allows the researcher to assess factors that determine the intensity of KBM. The intensity of each outcome variable is defined as the fraction of the number of correct answers provided by respondents for the sets of questions used in the survey and is estimated by the MVFP by treating those answers as a fractional outcome variable (Murteira and Ramalho, 2016). The MVFP allows the interdependence of the KBM outcome variables.

Because knowledge (*K*), belief (*B*), and management (*M*) are not directly observable, they can be represented by latent variables, and, which underlie the knowledge, belief, and management status of decision-making units in the sample. Following Schwiebert, (2018), the relationship between the unobservable latent variable (e.g., *K^*^_s_*) and the outcome of interest (e.g., *K_s_*) can be specified as follows;

*K*s=βk0+βk1X1…………**, …………………………………………………………* (9)

*B^*^_s_=β^b^_0_+β^b^_1_X_1_…………+**…………………………………………………………..* (10)

*M^*^_s_=β^m^_0_ +β^m^_1_ X_1_………+**…………………………………………………………..* (11)

where 𝛽 and 𝑋_𝑛_ aew as previously defined, 𝐾_𝑠_, 𝐵_𝑠_ and 𝑀_𝑠_ are fractional dependent variables that describe the share of total score obtained by the household, and 𝑒^𝑘^, 𝑒^𝑏^, and 𝑒^𝑚^ are disturbance terms assumed to be independent and identical across individual households (Murteira and Ramalho, 2016). The error term, 𝑒 = (𝑒^𝑘^, 𝑒^𝑏^, 𝑒^𝑚^) is multivariate normally distributed with a mean vector of zeros and a correlation matrix (Schwiebert, 2018):

 ………………………………………………………. (12)

In this study, the unknown parameters 𝛽 and 𝜌 were estimated using a seemingly unrelated regression with ordered responses (Greene and Hencher, 2010) under the conditional mixed process estimator with multilevel random effects command “cmp” available in STATA software.

## ***2.3.Study Area and Sampling Procedure***

This study was carried out in the three ADs hotspot counties of Baringo, Kilifi, and Kwale in

Kenya (Figure 1). Baringo is prone to floods leading to outbreaks of arboviral diseases. For instance, the 1997/98 El-Niño rains resulted in an episode of yellow fever, while the 2006/07 heavy rains resulted in an outbreak of RVF (Nguku *et al*., 2010). Kwale and Kilifi are areas from where the chikungunya virus started before spreading to other parts of the country, representing one of the critical seeding regions for ADs. Malaria, dengue fever, chikungunya fever, and lymphatic filariasis are common mosquito-borne diseases in the two areas (Ndenga *et al*., 2017).

## [Insert Figure 1 here]

Initially, focus group discussions were conducted in the study sites to determine the most important ADs and to adjust the survey tool. According to community members living in the three study areas, RVF, Chikungunya fever, and Dengue fever were the most prevalent ADs. Later, a multistage sampling technique was used to select 629 respondents for a survey of their KBM of ADs in their locale. In the first stage, the three ADs hotspot counties (Baringo, Kilifi, and Kwale) were purposively selected. In the second stage, purposive sampling was also used to select the most ADs-prone sub-counts in each of the three counties resulting in three study sites of Marigat in Baringo, Malindi in Kilifi and Msambweni in Kwale. A sampling frame of all households in the three study sites was obtained from the local administration (chiefs and village elders). In the third stage, a simple random sampling technique was used to select 200 households from each study site giving a total sample of 629 households after adjusting for 10 percent of the non-responses following Mutiso (2018). Well-trained enumerators undertook faceto-face interviews through a pre-tested semi-structured questionnaire using CSPro electronic data collection software.

### *2.4.Measurement of Variables*

#### 2.4.1. Outcome variables

The outcome variables of interest in this study included knowledge, beliefs, and management practices of ADs. These variables were measured as dummy variables. The knowledge score was constructed using 55 binary response questions for RVF and 14 each for chikungunya and dengue fevers. A total of 8 and 7 binary response questions were used to generate the beliefs and management scores for the three ADs. The beliefs section consisted of the perceived threat associated with ADs. The management practices were related to a group of actions taken to prevent the spread of ADs (Higuera-Mendieta *et al*., 2016). A respondent was considered knowledgeable, to have positive beliefs, and as having good ADs management practices of the three ADs when they correctly answered 50 percent of the questions posed under each outcome (*KBM*) variable. Based on this, the outcome variables took a value of one if the respondent answered 50 percent of the questions correctly and zero otherwise (i.e., having either an incorrect response, answering “I don’t know”, or having missing answers). The fractional variable used in the MVFP model was constructed as the sum of correct answers to knowledge, belief, and management practices questions as a ratio of the total number of questions asked per outcome variable. For instance, the intensity of knowledge of RVF was measured as the number of correct answers as a share of the 55 knowledge questions of RVF. The intensity of knowledge of dengue and chikungunya fever were measured as the number of correct answers as a share of 14 questions, Moreover, the intensity of beliefs on each of the three ADs was measured as a ratio of the correct answers to a total 8 questions. Finally, the intensity of management of the three ADs was measured as the number of correct answers as a share of 7 questions.

#### 2.4.2. Explanatory variables

The choice of the explanatory variables used in this study was informed by previous studies

(Abdi *et al*., 2015; Affognon *et al*., 2017; Dhimal *et al*., 2014; Harapan *et al*., 2018; Mallhi *et al*., 2018). These variables included access to health information, social capital and networks, asset endowment and household demographic characteristics.

Three variables were used to measure *“access to health information”*, distance to the nearest health facility, awareness of health impacts of ADs, and household experience with an AD. The distance between the homestead and the nearest health facility measured in the amount of time it takes to walk between the two points was used as proxy for access to health information. The variable awareness of health impacts took a value of one if the respondent understood the health impacts of ADs, and zero otherwise. Experience with an AD was measured as a dummy variable with a value of one if a family member had suffered from any AD in the 12 months preceding the survey and zero otherwise. Social capital and networking was proxied by group membership measured as a dummy variable with the value of 1 if the respondent was a member of a health promotion group and 0 otherwise. The number of tropical livestock units (TLU) kept by a household was used as a proxy for asset ownership in this study. The heterogeneity of the households was controlled in the regression model by including the household head’s education level, gender, and religion. The level of formal education attained was measured as the number of years of formal schooling completed by the household head. The gender of the household head was measured as a dummy variable, taking a value of one if the household head was male and zero otherwise. In this study, religion was measured as a categorical variable coded 0, 1 and 2 for other religions, Christianity, and Islam, respectively.

### 3. Results

#### 3.1. Descriptive Results

Table 1 presents the socioeconomic characteristics of households in the three ADs hotspots in Kenya. Overall, 55 percent of the households were aware of the health impacts of ADs while 33 percent of the households had suffered from at least one of them. Christianity was the dominant religion, as reported by 63 percent of the respondents, even though almost all respondents (98 percent) in Kwale were Muslims. On the average, Baringo and Kilifi residents took longer (37 and 35 minutes respectively) to reach the nearest health facility as compared to Kwale residents (24 minutes). Eleven percent of Kwale households belonged to a health promotion group as compared to five percent in the other two study sites. The average number of livestock owned in the study area was 3.69.

## [Insert Table 1 here]

Table 2 presents summary statistics of farmers KBM of ADs in Kenya. On the whole, 16, 29, and 18 percent of respondents had good knowledge of RVF, chikungunya fever, and dengue fever infections, respectively. The highest knowledge of RVF was recorded in Baringo (20 percent), while Kwale (36 percent) and Kilifi (23 percent) residents had the highest knowledge of chikungunya fever and dengue fever, respectively. Despite the low knowledge, most respondents had positive beliefs about the three ADs. In Kwale County, 75 and 55 percent of respondents believed that chikungunya and dengue fever respectively were dangerous diseases as compared to 44 and 38 percent of the respondents in Kilifi County. The low level of knowledge in the study areas translated into poor management of ADs that ranges between 6 percent in RVF and 27 percent of chikungunya fever.

## [Insert Table 2 here]

Tables 3 presents farmer’s specific knowledge of signs, symptoms, transmission methods, beliefs and management practices of the three ADs in Kenya. The main sign of the three ADs in human were fever, abdominal pains and headache as reported by 82, 29 and 19 percent of the respondents, respectively. In animals, the major signs of RVF were bloody diarrhea, bloody discharge and death among young ones as reported by 58, 57 and 56 percent of the respondents. Over 80 percent of respondents correctly identified mosquitoes as the vectors of the three diseases. Direct contact with blood and other body tissues of infected animals/humans were reported as common methods of transmission of RVF and dengue fever by 48 and 13 percent of the respondents respectively. When asked about the *Aedes* mosquito’s breeding grounds, less than 40 percent of the respondents indicated that mosquitoes breed in water containers. A majority of respondents did not know when the *Aedes* mosquito bites and incorrectly identified nighttime as the biting time. In comparison, less than two percent of the respondents correctly indicated that chikungunya and dengue vectors bite during the day.

## [Insert Table 3 here]

Mosquito nets were the most widely used method of preventing mosquito bites as reported by over 90 percent of the respondents. Over half of the respondents reported bush clearing as the most prevalent method used in the control of mosquito breeding, whereas covering water-holding containers and/or their proper disposal was reported by only a quarter of the respondents. Eighty and 64 percent of respondents respectively, reported that the management of RVF was the responsibility of the Veterinary Department and the Ministry of Health. Treatment in hospitals was the most dominant management practice followed by purchasing drugs in pharmaceutical outlets, using traditional treatment and using local herbs.

### 3.2. Econometric results

The correlation coefficients of the error terms of the multivariate probit MLE estimates of the drivers of KBM of ADs in Kenya was assessed (Table 4). The likelihood ratio rejects the null hypothesis of no correlation between the three equations’ error terms. This confirms the use of the MVP model instead of binary choice models. Some of the pair-wise correlation coefficients between the error terms in the KBM equations are significant, which further supports the MVP model. Knowledge complements beliefs in all three diseases. We estimated MVP and MVFP specifications and find the statistically significant variables in both models to be the same. For brevity, we only present the results of the MVFP model.

## [Insert Table 4 here]

The Multivariate Fractional Probit (MVFP) maximum likelihood estimates (MLE) of the intensity of farmers KBM of ADs in Kenya was calculated (Table 5). Access to information (experience and awareness), income, and some household characteristics (education, and religion) positively and significantly influence the intensity of farmers KBMs on ADs at least at the 5 percent level. In conformity with the expectations, awareness of the health impacts of ADs positively influenced the intensity of farmers KBM of all three ADs in Kenya except for the management of RVF and was significant at least at the 5 percent level. Income had positive significant influences on the intensity of KMB of the three ADs in Kenya at least at the 5 percent level except for the case of the beliefs on RVF.

Moreover, education positively influenced the intensity of farmer’s knowledge and management practices of the three ADs with the exception of the dengue fever and was significant at least at the 5 percent level. Belonging to the Muslim faith positively influenced the intensity of farmers’ beliefs on RVF and was significant at the 1 percent level. While being a Christian reduced the intensity of farmer’s beliefs regarding dengue fever, being a Muslim increased the intensity of farmers’ beliefs towards RVF. However, belonging to a health group and the distance to the nearest health facility negatively influence the intensity of farmers’ beliefs and management practices of dengue and RVF respectively and were significant at the 5 percent level.

## [Insert Table 5 here]

### 4. Discussion

Slightly above half of the households in the study were aware about ADs while a third of them had experienced the ADs (a family member had suffered from one of the ADs). On the one hand, a majority of the households in Baringo were aware about RVF but none of them was aware about the health impacts of chikungunya and dengue fever. On the other hand, 51 percent of the households in Kwale and Kilifi Counties were aware of the health impacts of chikungunya and dengue fever. More Baringo residents experienced RVF infections among family members as compared to Kilifi and Kwale counties. Similarly, more households in Kwale and Kilifi had family members who had suffered from chikungunya and dengue fever as compared to households in Baringo County. These findings support earlier studies that have reported RVF to be endemic in Baringo County while Kwale and Kilifi counties are hotspots of both chikungunya and dengue fever.

With regard to the KBM, we find that 88 percent of the respondents had poor knowledge about the three ADs. However, a majority of the households believed that the three ADs were dangerous diseases. Good management skills of the three ADs were reported by between 6 and 27 percent of the respondents. Fever was the most prevalent sign and symptom of ADs among humans. This is consistent with other studies that report fever as the most frequently stated symptom of RVF (Abdi *et al*., 2015), chikungunya fever (Bedoya-Arias *et al*., 2015), and dengue fever (Dhimal *et al*., 2014). Other disease signs and symptoms mentioned by less than 30 percent of the respondents included abdominal pains and headaches among humans and bloody diarrhea, bloody discharge and death of young ones among animals. These findings suggest that most of the respondents had limited knowledge of these AD signs, symptoms, and methods used to control their spread.

The most widely used methods of mosquito control reported by respondents in declining order of importance included use of nets, bush clearing, covering water-holding containers and/or their proper disposal. However, mosquito nets may offer little protection in reducing the risk of ADs

(Tsuzuki *et al*., 2010; Weaver, 2013). Similar findings have been reported in Kenya (Abdi *et al*., 2015) and Pakistan (Mallhi *et al*., 2018). Lack of such knowledge, especially in areas with a high density of A. *aegypti,* poses a challenge in ADs prevention. Almost all respondents reported that management of ADs was the responsibility of the Veterinary Department and the Ministry of Health. Expecting that government departments would control ADs might hinder communitybased efforts towards controlling their spread leading to increased risk of infection. Bartumeus *et al.,* (2019) highlight the importance of local communities in vector control. These findings are however consistent with other studies that have reported hospital health-seeking behaviour among households in managing ADs (Kumaran *et al*., 2018; Nguyen *et al*., 2019).

We find positive significance influences of access to information (experience and awareness), income, education, distance to a health facility and religion on the intensity of farmers KBM of

ADs in Kenya. Consistent with previous studies (Higuera-Mendieta *et al*., 2016; Kumaran *et al*., 2018; Nguyen *et al*., 2019), household education level increases the intensity of knowledge, beliefs and management of ADs. Information access (experience and awareness) increased farmers intensities of KBM of ADs in Kenya. Awareness of the health impact of the three ADs increased the intensity of their KBM by between 31 and 154 percent. Households that had experienced RVF and chikungunya fever had a higher intensity of knowledge and beliefs of both diseases by between 8 and 54 percent. This is consistent with other studies (Abdi *et al*., 2015; Harapan *et al*., 2018). Furthermore, experience and awareness of health impacts are important in influencing the management of ADs. Individuals who are aware of AD health impacts are more likely to undertake disease mitigation strategies or seek medical intervention (Sørensen *et al*., 2012).

Income was associated with increased farmer intensities of KBM on ADs in Kenya. A 1 percentage increase in household income increased livestock farmers intensities of KBM of the three ADs by between 4 and 13 percent. A direct relationship between income and good knowledge of dengue has been documented (Azfar *et al*., 2017; Lugova and Wallis, 2017; Selvarajoo *et al*., 2020). Similarly, Alhoot *et al*. (2017), Ghani *et al*. (2019), and Lugova and Wallis (2017) have reported a significant association between income and positive beliefs regarding dengue fever. The possible reason for a positive association between the intensity of

KBM and income is that people with higher economic status might have better information access on ADs and resources to manage the diseases (Castro *et al*., 2013; Selvarajoo *et al*., 2020).

An extra year spent in school increased a farmer’s intensity of management practices of RVF and chikungunya fever by 2 percent while that of dengue fever increased by 3 percent. Education improves access to information and provides individuals with the ability to interpret and implement different disease management strategies (Harapan *et al*., 2018). The relationship between education and management of ADs has been documented in other studies (Dhimal *et al*., 2014; Harapan *et al*., 2018; Naing *et al*., 2011; Nguyen *et al*., 2019). Education provides good knowledge of disease signs and symptoms as illustrated by Khun and Manderson (2007) which is important for timely disease prevention.

Farmers who were located further away from health facilities had poor management skills of ADs as compared to their counterparts who had better access to medical facilities. An extra minute spend walking to the health facility to seek treatment reduced the intensity of a farmers management skills of RVF by 6 percent. Feikin *et al*. (2009) has documented a negative relationship between distance of residence from a health facility and utilisation of health services in Kenya. Health facilities are the principal point for sourcing health information in many rural settings through the distribution of education materials on signs and symptoms and prevention methods of a diseases (He *et al*., 2016).

Being a Muslim increased the intensity of farmers’ beliefs that RVF is a dangerous disease by a huge margin. Similar finding has been documented by Chandren *et al.,* (2015) and Harapan *et al*.

(2018) which indicated that religion influenced people’s beliefs regarding dengue fever in Malaysia and Indonesia. Religion influences people’s beliefs regarding their willingness to receiving a certain treatment (Bousso *et al*., 2010).

### 5. Conclusions and policy implications

This study evaluates livestock farmer’s knowledge, beliefs, and management of RVF, chikungunya, and dengue fever in Kenya using a MVFP model that employs a sample of 629 households. Slightly above half of the respondents were aware of the three ADs, while a third of the respondents had experienced (suffered) the ADs. While only a small share of the respondents have basic knowledge about the three diseases, a vast majority of them considered ADs as serious diseases affecting both animals and humans. Despite the low knowledge, more than half of the respondent expressed positive beliefs towards ADs. There was a low translation of knowledge about disease transmission and prevention into good management practices.

The empirical results show that access to information (experience, awareness and distance to a health facility), income, education, and religion significantly influenced the intensity of farmers KBM of ADs in Kenya. This finding demonstrates the importance of access to information in influencing the KBM of ADs. Thus, policy initiatives should focus on increasing farmers’ awareness of the three ADs in Kenya to mitigate their negative health impacts. Moreover, the awareness programs on these three ADs should also target different religions separately. Most importantly, ADs prevention and control should be promoted among individuals who have experienced the diseases, their families, and visiting neighbors by hospitals to raise awareness among community members and use them as outreach program. This will increase the knowledge of ADs’ and improve the management of these diseases in the society.

### 6. References

Abdi, I. H., Affognon, H. D., Wanjoya, A. K., Onyango-Ouma, W., and Sang, R. (2015). Knowledge,

Attitudes and Practices (KAP) on Rift Valley Fever among Pastoralist Communities of Ijara District, North Eastern Kenya. *PLoS Neglected Tropical Diseases*, *9*(11). https://doi.org/10.1371/journal.pntd.0004239

Aerts, C., Revilla, M., Duval, L., Paaijmans, K., Chandrabose, J., Cox, H., and Sicuri, E. (2020). Understanding the role of disease knowledge and risk perception in shaping preventive behavior for selected vector-borne diseases in Guyana. *PLOS Neglected Tropical Diseases*, *14*(4), e0008149. https://doi.org/10.1371/journal.pntd.0008149

Affognon, H., Mburu, P., Hassan, O. A., Kingori, S., Ahlm, C., Sang, R., and Evander, M. (2017). Ethnic groups’ knowledge, attitude and practices and Rift Valley fever exposure in Isiolo County of Kenya. *PLoS Neglected Tropical Diseases*, *11*(3). https://doi.org/10.1371/journal.pntd.0005405

Ajzen, I. (2020). The theory of planned behavior: Frequently asked questions. *Human Behavior and Emerging Technologies*, *2*(4), 314–324.

Alahdal, H., Basingab, F., and Alotaibi, R. (2020). An analytical study on the awareness, attitude and practice during the COVID-19 pandemic in Riyadh, Saudi Arabia. *Journal of Infection and Public Health*, *13*(10), 1446–1452.

Alhoot, M. A., Baobaid, M. F., Al-Maleki, A. R., Abdelqader, M. A., Paveetran, A., Bathmanathan, L., Murugan, A., and Maniam, L. (2017). Knowledge, attitude, and practice towards dengue fever among patients in Hospital Taiping. *Malaysian Journal of Public Health Medicine*, *17*(3), 66–75.

Atoni, E., Waruhiu, C., Nganga, S., Xia, H., and Yuan, Z. (2018). Arboviruses of Human Health significance in Kenya. *African Journal of Health Sciences*, *31*(1), 121–141.

Azfar, M., Omarulharis, S., Azfar, H., Maryam, A., Hafizah, S., Adibah, B., and Akmal, N. (2017). Knowledge, attitude and practice of dengue prevention among sub urban community in Sepang, Selangor. *International Journal of Public Health and Clinical Sciences*, *4*(2), 73–83.

Bartumeus, F., Costa, G. B., Eritja, R., Kelly, A. H., Finda, M., Lezaun, J., Okumu, F., Quinlan, M. M., Thizy, D. C., Toé, L. P., and Vaughan, M. (2019). Sustainable innovation in vector control requires strong partnerships with communities. *PLOS Neglected Tropical Diseases*, *13*(4), e0007204. https://doi.org/10.1371/journal.pntd.0007204

Bedoya-Arias, J. E., Murillo-García, D. R., Bolaños-Muñoz, E., Hurtado-Hurtado, N., Ramírez-Jaramillo, V., Granados-Álvarez, S., and Rodriguez-Morales, A. J. (2015). Healthcare students and workers’ knowledge about epidemiology and symptoms of chikungunya fever in two cities of Colombia.

*The	Journal	of	Infection	in	Developing	Countries*,	*9*(03),	330–332. https://doi.org/10.3855/jidc.6445

Birol, E., Asare-Marfo, D., Ayele, G., Mensah-Bonsu, A., Ndirangu, L., Okpukpara, B., Roy, D., and Yakhshilikov, Y. (2013). The impact of avian flu on livelihood outcomes in Africa: Evidence from Ethiopia, Ghana, Kenya and Nigeria. *African Journal of Agricultural and Resource Economics*, *8*(311-2016–5566).

Bousso, R. S., Serafim, T. de S., and Misko, M. D. (2010). The relationship between religion, illness and death in life histories of family members of children with life-threatening diseases. *Revista Latino-Americana de Enfermagem*, *18*(2), 156–162.

Braack, L., Gouveia de Almeida, A. P., Cornel, A. J., Swanepoel, R., and de Jager, C. (2018). Mosquitoborne arboviruses of African origin: Review of key viruses and vectors. *Parasites & Vectors*, *11*(1), 29. https://doi.org/10.1186/s13071-017-2559-9

Caminade, C., McIntyre, K. M., and Jones, A. E. (2019). Impact of recent and future climate change on vector borne diseases. *Annals of the New York Academy of Sciences*, *1436*(1), 157. https://doi.org/10.1111/nyas.13950

Castro, M., Sánchez, L., Pérez, D., Sebrango, C., Shkedy, Z., and Stuyft, P. V. der. (2013). The

Relationship between Economic Status, Knowledge on Dengue, Risk Perceptions and Practices. *PLOS ONE*, *8*(12), e81875. https://doi.org/10.1371/journal.pone.0081875

Chandren, J. R., Wong, L. P., and AbuBakar, S. (2015). Practices of Dengue Fever Prevention and the Associated Factors among the Orang Asli in Peninsular Malaysia. *PLOS Neglected Tropical Diseases*, *9*(8), e0003954. https://doi.org/10.1371/journal.pntd.0003954

Chib, S., and Greenberg, E. (1998). Analysis of Multivariate Probit Models. *Biometrika*, *85*(2), 347–361.

Dente, M. G., Riccardo, F., Van Bortel, W., Marrama, L., Mollet, T., Derrough, T., Sudre, B., Calistri, P., Nacca, G., Ranghiasci, A., Escadafal, C., Gaayeb, L., Guillot, A., Jiménez-Clavero, M. A.,

Manuguerra, J.-C., Mikaty, G., Picard, M., Fernández-Pinero, J., Pérez-Ramírez, E., … Declich, S. (2020). Enhancing Preparedness for Arbovirus Infections with a One Health Approach: The

Development and Implementation of Multisectoral Risk Assessment Exercises. *BioMed Research International*, *2020*, e4832360. https://doi.org/10.1155/2020/4832360

Dhimal, M., Aryal, K. K., Dhimal, M. L., Gautam, I., Singh, S. P., Bhusal, C. L., and Kuch, U. (2014). Knowledge, Attitude and Practice Regarding Dengue Fever among the Healthy Population of Highland and Lowland Communities in Central Nepal. *PLOS ONE*, *9*(7), e102028. https://doi.org/10.1371/journal.pone.0102028

Ellis-Iversen, J., Cook, A. J. C., Watson, E., Nielen, M., Larkin, L., Wooldridge, M., and Hogeveen, H. (2010). Perceptions, circumstances and motivators that influence implementation of zoonotic control programs on cattle farms. *Preventive Veterinary Medicine*, *93*(4), 276–285.

https://doi.org/10.1016/j.prevetmed.2009.11.005

Feikin, D. R., Nguyen, L. M., Adazu, K., Ombok, M., Audi, A., Slutsker, L., and Lindblade, K. A. (2009). The impact of distance of residence from a peripheral health facility on pediatric health utilisation in rural western Kenya. *Tropical Medicine & International Health*, *14*(1), 54–61.

Fishbein, M., and Ajzen, I. (1977). Belief, Attitude, Intention, and Behavior: An Introduction to Theory and Research. *Philosophy and Rhetoric*, *10*(2), 130–132.

Ghani, N. A., Shohaimi, S., Hee, A. K.-W., Chee, H.-Y., Emmanuel, O., and Alaba Ajibola, L. S. (2019). Comparison of knowledge, attitude, and practice among communities living in hotspot and nonhotspot areas of dengue in Selangor, Malaysia. *Tropical Medicine and Infectious Disease*, *4*(1), 37.

Girard, M., Nelson, C. B., Picot, V., and Gubler, D. J. (2020). Arboviruses: A global public health threat. *Vaccine*, *38*(24), 3989–3994. https://doi.org/10.1016/j.vaccine.2020.04.011

Harapan, H., Rajamoorthy, Y., Anwar, S., Bustamam, A., Radiansyah, A., Angraini, P., Fasli, R., Salwiyadi, S., Bastian, R. A., Oktiviyari, A., Akmal, I., Iqbalamin, M., Adil, J., Henrizal, F., Darmayanti, D., Pratama, R., Setiawan, A. M., Mudatsir, M., Hadisoemarto, P. F., … Müller, R.

(2018). Knowledge, attitude, and practice regarding dengue virus infection among inhabitants of Aceh, Indonesia: A cross-sectional study. *BMC Infectious Diseases*, *18*(1), 96. https://doi.org/10.1186/s12879-018-3006-z

He, Z., Cheng, Z., Shao, T., Liu, C., Shao, P., Bishwajit, G., Feng, D., and Feng, Z. (2016). Factors

Influencing Health Knowledge and Behaviors among the Elderly in Rural China. *International Journal of Environmental Research and Public Health*, *13*(10), Article 10. https://doi.org/10.3390/ijerph13100975

Higuera-Mendieta, D. R., Cortés-Corrales, S., Quintero, J., and González-Uribe, C. (2016). KAP Surveys and Dengue Control in Colombia: Disentangling the Effect of Sociodemographic Factors Using Multiple Correspondence Analysis. *PLOS Neglected Tropical Diseases*, *10*(9), e0005016. https://doi.org/10.1371/journal.pntd.0005016

Kan, M. P. H., and Fabrigar, L. R. (2017). Theory of Planned Behavior. In V. Zeigler-Hill and T. K. Shackelford (Eds.), *Encyclopedia of Personality and Individual Differences* (pp. 1–8). Springer

International Publishing. https://doi.org/10.1007/978-3-319-28099-8_1191-1

Kassie, M., Teklewold, H., Jaleta, M., Marenya, P., and Erenstein, O. (2015). Understanding the adoption of a portfolio of sustainable intensification practices in eastern and southern Africa. *Land Use Policy*, *42*, 400–411.

Khanna, M. (2001). Sequential Adoption of Site-Specific Technologies and its Implications for Nitrogen Productivity: A Double Selectivity Model. *American Journal of Agricultural Economics*, *83*(1), 35–51. https://doi.org/10.1111/0002-9092.00135

Khun, S., and Manderson, L. (2007). Community and School-Based Health Education for Dengue Control in Rural Cambodia: A Process Evaluation. *PLoS Neglected Tropical Diseases*, *1*(3). https://doi.org/10.1371/journal.pntd.0000143

Kumaran, E., Doum, D., Keo, V., Sokha, L., Sam, B., Chan, V., Alexander, N., Bradley, J., Liverani, M., Prasetyo, D. B., Rachmat, A., Lopes, S., Hii, J., Rithea, L., Shafique, M., and Hustedt, J. (2018). Dengue knowledge, attitudes and practices and their impact on community-based vector control in rural Cambodia. *PLoS Neglected Tropical Diseases*, *12*(2), e0006268. https://doi.org/10.1371/journal.pntd.0006268

Lugova, H., and Wallis, S. (2017). Cross-sectional survey on the dengue knowledge, attitudes and preventive practices among students and staff of a public university in Malaysia. *Journal of Community Health*, *42*(2), 413–420.

Ma, J., Ye, X., and Shi, C. (2018). Development of multivariate ordered probit model to understand household vehicle ownership behavior in Xiaoshan District of Hangzhou, China. *Sustainability*, *10*(10), 3660.

Mallhi, T. H., Khan, Y. H., Tanveer, N., Bukhsh, A., Khan, A. H., Aftab, R. A., Khan, O. H., and Khan,

T. M. (2018). Awareness and knowledge of Chikungunya infection following its outbreak in Pakistan among health care students and professionals: A nationwide survey. *PeerJ*, *6*, e5481. https://doi.org/10.7717/peerj.5481

Mangesho, P. E., Neselle, M. O., Karimuribo, E. D., Mlangwa, J. E., Queenan, K., Mboera, L. E., Rushton, J., Kock, R., Häsler, B., and Kiwara, A. (2017). Exploring local knowledge and perceptions on zoonoses among pastoralists in northern and eastern Tanzania. *PLoS Neglected Tropical Diseases*, *11*(2), e0005345.

Marchi, S., Trombetta, C. M., and Montomoli, E. (2018). Emerging and Re-emerging Arboviral Diseases as a Global Health Problem. *Public Health; Majumder, MAA, Ed.; IntechOpen: London, UK*, 25– 46.

Maurice, A. de S., Nyakarahuka, L., Purpura, L., Ervin, E., Tumusiime, A., Balinandi, S., Kyondo, J., Mulei, S., Tusiime, P., Manning, C., Rollin, P. E., Knust, B., and Shoemaker, T. (2018). Rift Valley Fever: A survey of knowledge, attitudes, and practice of slaughterhouse workers and community members in Kabale District, Uganda. *PLOS Neglected Tropical Diseases*, *12*(3), e0006175. https://doi.org/10.1371/journal.pntd.0006175

Mingolla, C., Hudders, L., Vanwesenbeeck, I., and Claerebout, E. (2019). Towards a biased mindset: An extended Theory of Planned Behaviour framework to predict farmers’ intention to adopt a sustainable mange control approach. *Preventive Veterinary Medicine*, *169*, 104695. https://doi.org/10.1016/j.prevetmed.2019.104695

Munyua, P., Murithi, R. M., Wainwright, S., Githinji, J., Hightower, A., Mutonga, D., Macharia, J., Ithondeka, P. M., Musaa, J., Breiman, R. F., Bloland, P., and Njenga, M. K. (2010). Rift Valley

fever outbreak in livestock in Kenya, 2006-2007. *The American Journal of Tropical Medicine and Hygiene*, *83*(2 Suppl), 58–64. https://doi.org/10.4269/ajtmh.2010.09-0292

Muriithi, B. W., Menale, K., Diiro, G., and Muricho, G. (2018). Does gender matter in the adoption of push-pull pest management and other sustainable agricultural practices? Evidence from Western Kenya. *Food Security*, *10*(2), 253–272.

Murteira, J. M., and Ramalho, J. J. (2016). Regression analysis of multivariate fractional data. *Econometric Reviews*, *35*(4), 515–552.

Mutiso, J. (2018). *An assessment of factors affecting women’s behavior towards maternal, infant and young child feeding in Homa Bay County, Kenya.* [University of Nairobi]. http://erepository.uonbi.ac.ke/bitstream/handle/11295/103108/JANET%20MWENDE%20MUTI SO%20.pdf?sequence=1&isAllowed=y

Naing, C., Ren, W. Y., Man, C. Y., Fern, K. P., Qiqi, C., Ning, C. N., and Ee, C. W. S. (2011). Awareness of Dengue and Practice of Dengue Control Among the Semi-Urban Community: A Cross Sectional Survey. *Journal of Community Health*, *36*(6), 1044–1049. https://doi.org/10.1007/s10900-011-9407-1

Ndenga, B. A., Mutuku, F. M., Ngugi, H. N., Mbakaya, J. O., Aswani, P., Musunzaji, P. S., Vulule, J., Mukoko, D., Kitron, U., and LaBeaud, A. D. (2017). Characteristics of Aedes aegypti adult mosquitoes in rural and urban areas of western and coastal Kenya. *PLOS ONE*, *12*(12), e0189971. https://doi.org/10.1371/journal.pone.0189971

Nguku, P. M., Sharif, S. K., Mutonga, D., Amwayi, S., Omolo, J., Mohammed, O., Farnon, E. C., Gould, L. H., Lederman, E., Rao, C., Sang, R., Schnabel, D., Feikin, D. R., Hightower, A., Njenga, M. K., and Breiman, R. F. (2010). An investigation of a major outbreak of Rift Valley fever in Kenya: 2006-2007. *The American Journal of Tropical Medicine and Hygiene*, *83*(2 Suppl), 5–13. https://doi.org/10.4269/ajtmh.2010.09-0288

Nguyen, H. V., Than, P. Q. T., Nguyen, T. H., Vu, G. T., Hoang, C. L., Tran, T. T., Truong, N. T., Nguyen, S. H., Do, H. P., Ha, G. H., Nguyen, H. L. T., Dang, A. K., Do, C. D., Tran, T. H., Tran, B. X., Latkin, C. A., Ho, C. S. H., and Ho, R. C. M. (2019). Knowledge, Attitude and Practice about Dengue Fever among Patients Experiencing the 2017 Outbreak in Vietnam. *International Journal of Environmental Research and Public Health*, *16*(6), Article 6. https://doi.org/10.3390/ijerph16060976

Okello, G. B. A., Agata, N., Ouma, J., Cherogony, S. C., Tukei, P. M., Ochieng, W., Boer, J. W. D., and Sanders, E. J. (1993). Outbreak of yellowfever in Kenya. *The Lancet*, *341*(8843), 489. https://doi.org/10.1016/0140-6736(93)90237-B

Owange, N. O., Ogara, W. O., Affognon, H., Peter, G. B., Kasiiti, J., Okuthe, S., Onyango-Ouma, W., Landmann, T., Sang, R., and Mbabu, M. (2014). Occurrence of rift valley fever in cattle in Ijara district, Kenya. *Preventive Veterinary Medicine*, *117*(1), 121–128.

Rich, K. M., and Wanyoike, F. (2010). An Assessment of the Regional and National Socio-Economic Impacts of the 2007 Rift Valley Fever Outbreak in Kenya. *The American Journal of Tropical Medicine and Hygiene*, *83*(2 Suppl), 52–57. https://doi.org/10.4269/ajtmh.2010.09-0291

Schwiebert, J. (2018). *A sample selection model for fractional response variables*. Working Paper Series in Economics, No 382. Institute of Economics, Leuphana Universität Lüneburg.

Selvarajoo, S., Liew, J. W. K., Tan, W., Lim, X. Y., Refai, W. F., Zaki, R. A., Sethi, N., Sulaiman, W. Y. W., Lim, Y. A. L., and Vadivelu, J. (2020). Knowledge, attitude and practice on dengue prevention and dengue seroprevalence in a dengue hotspot in Malaysia: A cross-sectional study. *Scientific Reports*, *10*(1), 1–13.

Sergon, K., Njuguna, C., Kalani, R., Ofula, V., Onyango, C., Konongoi, L. S., Bedno, S., Burke, H., Dumilla, A. M., Konde, J., Njenga, M. K., Sang, R., and Breiman, R. F. (2008). Seroprevalence of Chikungunya virus (CHIKV) infection on Lamu Island, Kenya, October 2004. *The American Journal of Tropical Medicine and Hygiene*, *78*(2), 333–337.

Shabani, S. S., Ezekiel, M. J., Mohamed, M., and Moshiro, C. S. (2015). Knowledge, attitudes and practices on Rift Valley fever among agro pastoral communities in Kongwa and Kilombero districts, Tanzania. *BMC Infectious Diseases*, *15*(1), 1–9.

Sommer, L. (2011). The theory of planned behaviour and the impact of past behaviour. *International Business & Economics Research Journal (IBER)*, *10*(1), 91–110.

Song, X.-Y., and Lee, S.-Y. (2005). A multivariate probit latent variable model for analyzing dichotomous responses. *Statistica Sinica*, *15*(3), 645–664.

Sørensen, K., Van den Broucke, S., Fullam, J., Doyle, G., Pelikan, J., Slonska, Z., Brand, H., and (HLSEU) Consortium Health Literacy Project European. (2012). Health literacy and public health: A systematic review and integration of definitions and models. *BMC Public Health*, *12*(1), 80. https://doi.org/10.1186/1471-2458-12-80

Tsuzuki, A., Thiem, V. D., Suzuki, M., Yanai, H., Matsubayashi, T., Yoshida, L.-M., Minh, T. T., Anh, D. D., Kilgore, P. E., and Takagi, M. (2010). Can daytime use of bed nets not treated with insecticide reduce the risk of dengue hemorrhagic fever among children in Vietnam? *The American Journal of Tropical Medicine and Hygiene*, *82*(6), 1157–1159.

Vande Velde, F., Claerebout, E., Cauberghe, V., Hudders, L., Van Loo, H., Vercruysse, J., and Charlier, J. (2015). Diagnosis before treatment: Identifying dairy farmers’ determinants for the adoption of sustainable practices in gastrointestinal nematode control. *Veterinary Parasitology*, *212*(3), 308– 317. https://doi.org/10.1016/j.vetpar.2015.07.013

Weaver, S. C. (2013). Urbanization and geographic expansion of zoonotic arboviral diseases:

Mechanisms and potential strategies for prevention. *Trends in Microbiology*, *21*(8), 360–363.

Wensman, J. J., Lindahl, J., Wachtmeister, N., Torsson, E., Gwakisa, P., Kasanga, C., and Misinzo, G. (2015). A study of Rift Valley fever virus in Morogoro and Arusha regions of Tanzania–serology and farmers’ perceptions. *Infection Ecology & Epidemiology*, *5*(1), 30025.

https://doi.org/10.3402/iee.v5.30025

WHO. (2016). *“Yellow Fever – Kenya (Disease Outbreak News)".* Geneva, World Health Organization*.* http://www.who.int/csr/don/6-april-2016-yellow-fever-kenya/en/

WHO. (2020). *World Health Organization Fact Sheet: Vector-Borne Diseases*. Geneva, World Health Organization. https://www.who.int/news-room/fact-sheets/detail/vector-borne-diseases

Young, G., Valdez, E. A., and Kohn, R. (2009). Multivariate probit models for conditional claim-types.

*Insurance:	Mathematics	and	Economics*,	*44*(2),	214–228. https://doi.org/10.1016/j.insmatheco.2008.11.004

Figure	[Click here to access/download;Figure;Figures_PNTD.docx](https://www.editorialmanager.com/pntd/download.aspx?id=1013481&guid=70d0908c-1079-493a-9977-f17996d4b4be&scheme=1)

## ***Figure 1: Study area and sampled households***

Source: *icipe* GIS unit

Figure	[Click here to access/download;Table;Tables_PNTD.docx](https://www.editorialmanager.com/pntd/download.aspx?id=1013406&guid=a6ad0f80-6452-4c69-bdcc-2fad58f3c3b6&scheme=1)

**Table 1. Characteristics of Livestock Keepers in Kenya’s ADs Hotspots**

| ***Variables*** | | | **Baringo** | **Kwale** | **Kilifi** | **Overall** | |
| --- | --- | --- | --- | --- | --- | --- | --- |
|  | | | **n=211** | **n=218** | **n=200** | **n=629** | |
| *Household demographic characteristics* | | |  |  |  |  | |
| Education Household head’s years of formal education | | | 7.06^a^ | 6.50^a^ | 7.09^a^ | 6.88 | |
|  | | | (4.52) | (4.02) | (4.04) | (4.20) | |
| Gender Sex of the household head (1=male 0=female) | | | 0.85^a^ | 0.78^a^ | 0.83^a^ | 0.82 | |
| Religion Religion of respondent (1=Yes, 0=No)  Others | | | 0^a^ | 0^a^ | 0.05^b^ | 0.02 | |
| Christianity | | | 0.99^c^ | 0.02^a^ | 0.92^b^ | 0.63 | |
| Islam | | | 0^a^ | 0.97^c^ | 0.04^b^ | 0.35 | |
| *Access to health information* | | |  |  |  |  | |
| Experience | | Household suffered from RVF (1=Yes, 0=No) | 0.43^b^ | 0.00^a^ | 0.06^a^ | 0.30 | |
|  | | Household suffered from chikungunya fever (1=Yes, 0=No) | 0.00^a^ | 0.50^a^ | 0.43^a^ | 0.47 | |
|  | | Household suffered from dengue fever (1=Yes, 0=No) | - | 0.21^a^ | 0.25^a^ | 0.23 | |
| Awareness | | Household aware of RVF health impacts (1=Yes, 0=No) | 0.87^c^ | 0.00^a^ | 0.25^b^ | 0.63 | |
|  | | Household aware of health impacts of chikungunya fever (1=Yes, 0=No) | 0.00^ab^ | 0.71^b^ | 0.50^a^ | 0.62 | |
|  | | Household aware of health impacts of dengue fever (1=Yes, 0=No) | - | 0.45^a^ | 0.31^a^ | 0.40 | |
| Distance | | Distance to the nearest health facility (Walking minutes) | 36.50^b^ | 24.32^a^ | 34.61^b^ | 31.67 | |
|  | |  | (35.91) | (25.84) | 25.84 | 30.05 | |
| ***Social capital and networking*** | | |  |  |  |  | |
| Group Whether a member of the household belongs membership to a health promotion group (1=Yes 0=No) | | | 0.05^a^ | 0.11^b^ | 0.05^a^ | 0.07 | |
| ***Asset endowment*** | | |  |  |  |  | |
|  | Livestock | Livestock ownership in Tropical livestock unit (TLU) | 8.36^b^ | 1.36^a^ | 1.29^a^ | 3.69 |  |
|  |  |  | (13.28) | (6.16) | (1.87) | (9.18) |  |
|  | Income | Total income from all enterprises (KES/Year) 000 | 112.62^a^ | 169.50^a^ | 145.23^a^ | 142.70 |  |
|  |  |  | (142.93) | (443.67) | (264.76) | (312.44) |  |

*Notes:* Standard deviation in parenthesis; 1US$ = KES 102 at the survey time; Means in the same row, followed by the same letters, are not significantly different at 5%.

**Table 2: Farmers Knowledge, Beliefs and Management of ADs in Kenya**

| **Variable** | | **Description** | **Baringo** | **Kwale** | **Kilifi** | **Overall** | |
| --- | --- | --- | --- | --- | --- | --- | --- |
| **Outcome variables** | | *Dummy (1=yes if half of the number of questions were correctly answered*) |  |  |  |  | |
| **RVF** | |  | **n=207** | **n=23** | **n=89** | **n=319** | |
| Knowledge | | Knowledgeable of RVF | 0.20^b^ | 0.00^a^ | 0.09^a^ | 0.16 | |
| Beliefs | | Positive beliefs towards RVF | 0.88^c^ | 0.00^a^ | 0.33^b^ | 0.66 | |
|  | Management Have good management practices to prevent  RVF | | 0.04^a^ | 0.09^a^ | 0.09^a^ | 0.06 |  |
|  | **Chikungunya fever** | | **n=1** | **n=191** | **n=145** | **n=337** |  |
|  | Knowledge | Knowledgeable of Chikungunya fever | 0.00ab | 0.36^b^ | 0.19^a^ | 0.29 |  |
|  | Beliefs | Positive beliefs towards Chikungunya fever | 0.00ab | 0.75^b^ | 0.55^a^ | 0.66 |  |
|  | Management | Have good management practices to prevent Chikungunya fever | 0.00ab | 0.35^b^ | 0.17^a^ | 0.27 |  |
|  | **Dengue fever** |  | **n=0** | **n=84** | **n=52** | **n=136** |  |
|  | Knowledge | Knowledgeable of Dengue fever | - | 0.15^a^ | 0.23^a^ | 0.18 |  |
|  | Beliefs | Positive beliefs towards Dengue fever | - | 0.44^a^ | 0.38^a^ | 0.42 |  |
|  | Management | Have good management practices to prevent Dengue fever | - | 0.23^a^ | 0.17^a^ | 0.21 |  |

*Notes:* Standard deviation in parenthesis; Means in the same row, followed by the same letters, are not significantly different at 5%.

# Table 3: Farmers Knowledge of Signs, Symptoms, Transmission Methods, Beliefs and Management of ADs in Kenya

| **Characteristics** | | **RVF** | |  | **Chikungunya**  **fever** | | | **Dengue fever** | | | **Overall** | |
| --- | --- | --- | --- | --- | --- | --- | --- | --- | --- | --- | --- | --- |
|  |  | Number of observations (N) | | % | N % | | | N % | | | % | |
|  | Have heard about the disease | 629 | 51 | | 629 | 54 | | 629 | | 22 | 42 |  |
|  | Main signs and symptoms in humans |  |  | |  |  | |  | |  |  |  |
|  | Fever | 250 | 72 | | 273 | 85 | | 71 | | 89 | 82 |  |
|  | Generalized weakness | 250 | 43 | |  |  | |  | |  | 43 |  |
|  | Bleeding from nose and gums | 250 | 11 | |  |  | |  | |  | 11 |  |
|  | Skin rashes |  |  | |  |  | | 71 | | 10 | 10 |  |
|  | Back pain | 250 | 25 | |  |  | |  | |  | 25 |  |
|  | Nausea/vomiting | 250 | 8 | |  |  | | 71 | | 44 | 26 |  |
|  | Joint pain |  |  | | 273 | 32 | |  | |  | 32 |  |
|  | Abdominal pain | 207 | 28 | | 272 | 25 | | 71 | | 34 | 29 |  |
|  | Pain behind the eyes | 250 | 2 | | 272 | 12 | | 71 | | 8 | 7 |  |
|  | Abortion in pregnant women | 250 | 10 | |  |  | |  | |  | 10 |  |
|  | Inflammation of brain-headaches, coma, seizures | 250 | 20 | | 272 | 13 | | 71 | | 24 | 19 |  |
|  | Fatigue |  |  | | 272 | 34 | |  | |  | 34 |  |
|  | Main signs and symptoms in animals |  |  | |  |  | |  | |  |  |  |
|  | Abortion | 249 | 33 | |  |  | |  | |  | 33 |  |
|  | Bloody Discharge | 249 | 57 | |  |  | |  | |  | 57 |  |
|  | High fever | 249 | 54 | |  |  | |  | |  | 54 |  |
|  | Bloody Diarrhea | 249 | 58 | |  |  | |  | |  | 58 |  |
|  | Death Among young animals | 249 | 56 | |  |  | |  | |  | 56 |  |
|  | Is mosquito responsible for ADs transmission | 165 | 82 | | 200 | 96 | | 54 | | 94 | 91 |  |
|  | Direct contact with blood and other body tissues from an infected person/animal | 249 | | 48 |  |  | 71 | | 13 | | 31 |  |
|  | Do mosquitoes breed in water containers | 319 | | 29 | 337 | 35 | 136 | | 29 | | 31 |  |
|  | When are the *Aedes* mosquitoes most likely to feed/bite? |  | |  |  |  |  | |  | |  |  |
|  | Nighttime | 319 | | 59 | 337 | 77 | 136 | | 75 | | 70 |  |
|  | Day time | 319 | | 1 | 337 | 0 | 136 | | 1 | | 1 |  |
|  | Both day and night | 319 | | 39 | 337 | 23 | 136 | | 24 | |  |  |
|  | Awareness of the methods to prevent mosquito breeding |  | |  |  |  |  | |  | |  |  |
|  | Clearing bushes around the house | 319 | | 58 | 336 | 62 | 136 | | 60 | | 60 |  |
|  | Creating proper drainage of water around the home | 319 | | 31 | 336 | 40 | 136 | | 46 | | 39 |  |
|  | Covering water holding containers tightly | 319 | | 20 | 336 | 28 | 136 | | 32 | | 27 |  |
|  | Proper disposal of discarded containers | 319 | | 16 | 336 | 25 | 136 | | 23 | | 21 |  |
|  | Methods used to control mosquito bites |  | |  |  |  |  | |  | |  |  |
|  | Mosquito bed nets | 319 | | 96 | 336 | 98 | 136 | | 99 | | 98 |  |
|  | Mosquito repellants | 319 | | 38 | 336 | 53 | 136 | | 58 | | 50 |  |
|  | Indoor residual insecticides spraying | 319 | | 35 | 336 | 52 | 136 | | 65 | | 51 |  |
|  | Screening/fencing windows and doors | 319 | | 15 | 336 | 37 | 136 | | 43 | | 32 |  |
|  | Close doors and windows by 6.00PM | 319 | | 44 | 336 | 38 | 136 | | 35 | | 39 |  |
|  | Plants to repel Mosquitoes | 319 | | 6 | 336 | 10 | 136 | | 8 | | 8 |  |
|  | Player of a major role in the control of this disease |  | |  |  |  |  | |  | |  |  |
|  | Veterinary Authority | 249 | | 80 | 272 | 4 | 71 | | 1 | | 28 |  |
|  | Health Authority | 249 | | 64 | 272 | 90 | 71 | | 97 | | 84 |  |
|  | Environmental Authority | 249 | | 7 | 272 | 16 | 71 | | 17 | | 13 |  |
|  | Community | 249 | | 29 | 272 | 24 | 71 | | 32 | | 28 |  |
|  | What would your household do if you suspect that you or your family member has been infected with this disease? |  | |  |  |  |  | |  | |  |  |
|  | Local herb (e.g. pawpaw leaves to treat Chikungunya) | 250 | | 6 | 273 | 24 | 71 | | 1 | | 10 |  |
|  | Chemist medicine | 250 | | 5 | 273 | 7 | 71 | | 6 | | 6 |  |
|  | Seek traditional treatment | 250 | | 8 | 273 | 8 | 71 | | 4 | | 7 |  |
|  | Seek treatment in hospital | 250 | | 85 | 273 | 88 | 71 | | 92 | | 88 |  |

**Table 4. Correlation Coefficients of the Error Terms of the MVP Estimates**

**Disease**	*ρ*K	*ρ*B	*ρ*M

RVF	*ρ*K	1	*ρ*B	0.640 (0.136)**	1	*ρ*M	-0.371 (0.216)	-0.171 (0.190)	1

Likelihood ratio test of *ρ*KB = *ρ*KM = *ρ*BM =0: 𝜒^2^(3) = 8.500, Prob > 𝜒^2^ = 0.037

Chikungunya fever

*ρ*K	1	*ρ*B	0.364 (0.114)**	1	*ρ*M	0.538 (0.084)***	0.233 (0.111)**	1

Likelihood ratio test of *ρ*KB = *ρ*KM = *ρ*BM = 0: 𝜒^2^(3) = 42.558, Prob > 𝜒^2^ =0.000

Dengue fever	*ρ*K	1	*ρ*B	0.650 (0.165)**	1	*ρ*M	0.253(0.173)	0.216 (0.173)	1

Likelihood ratio test of *ρ*KB = *ρ*KM = *ρ*BM = 0: 𝜒^2^(3) = 7.002, Prob > 𝜒^2^ =0.072

Notes: Standard errors in parenthesis; K=Knowledge, B=Beliefs, M=Management; * = significant at p < 0.1; ** = significant at p < 0.05; *** = significant at p < 0.00

**Table 5: Multivariate Fractional Probit Maximum Likelihood Estimates of the Intensity of Farmers KBM of ADs in Kenya**

**RVF	Chikungunya fever	Dengue fever**

**^Variables^	Knowledge	Beliefs	Management	Knowledge	Beliefs	Management	Knowledge	Beliefs	Management**

| *Household demographic characteristics* |  |  |  |  |  |  |  |  |
| --- | --- | --- | --- | --- | --- | --- | --- | --- |
| Education 0.010* | 0.006 | 0.024*** | 0.006 | -0.015 | 0.024*** | 0.004 | -0.005 | 0.031** |
| (0.006) | (0.009) | (0.006) | (0.005) | (0.010) | (0.007) | (0.010) | (0.023) | (0.012) |
| Gender -0.097* | -0.045 | -0.112 | -0.054 | -0.030 | -0.080 | -0.007 | -0.190 | -0.077 |
| (0.054) | (0.115) | (0.070) | (0.043) | (0.091) | (0.060) | (0.084) | (0.204) | (0.090) |
| Christianity -0.165 | 0.325 | -0.207 | -0.086 | -0.060 | 0.054 | -0.045 | -0.768* | -0.015 |
| (0.209) | (0.309) | (0.170) | (0.184) | (0.207) | (0.122) | (0.378) | (0.417) | (0.198) |
| Islam 0.138 | 5.156*** | -0.130 | -0.112 | -0.390 | -0.066 | -0.619 | -0.279 | -0.089 |
| (0.215)  *Access to health information* | (0.370) | (0.181) | (0.217) | (0.265) | (0.138) | (0.379) | (0.442) | (0.232) |
| Experience 0.230*** | 0.240** | 0.079 | 0.080** | 0.547*** | -0.069 | 0.092 | 0.513** | 0.036 |
| (0.044) | (0.082) | (0.066) | (0.038) | (0.077) | (0.052) | (0.099) | (0.161) | (0.099) |
| Awareness 0.314*** | 0.762*** | 0.110 | 0.365*** | 0.739*** | 0.339** | 0.482*** | 1.544*** | 0.414*** |
| (0.073) | (0.103) | (0.098) | (0.041) | (0.090) | (0.058) | (0.080) | (0.180) | (0.087) |
| Distance 0.012 | -0.050 | -0.061** | -0.015 | -0.018 | -0.034 | 0.066* | 0.110 | 0.021 |
| (0.021)  *Social capital and networks* | (0.035) | (0.031) | (0.019) | (0.043) | (0.028) | (0.036) | (0.083) | (0.045) |
| Group membership -0.003 | 0.060 | 0.078 | -0.011 | 0.150 | -0.051 | -0.083 | -0.472** | -0.011 |
| (0.102)  *Asset endowment* | (0.162) | (0.126) | (0.066) | (0.154) | (0.100) | (0.080) | (0.208) | (0.112) |
| Livestock units 0.022 | -0.005 | -0.011 |  |  |  |  |  |  |
| (0.018) | (0.030) | (0.022) |  |  |  |  |  |  |
| Income 0.046** | 0.058 | 0.126*** | 0.048*** | 0.123*** | 0.041** | 0.067** | 0.213** | 0.084** |
| (0.020)  *Location fixed effects* | (0.038) | (0.026) | (0.012) | (0.034) | (0.017) | (0.029) | (0.071) | (0.034) |

(0.138)	(0.111)	(0.090)	(0.504)	(0.124)

Kwale

-

0.988***

-

6.859***

0.000

-

0.284**

6.324***

0.199

(0.126)

(0.434)

(0.229)

(0.140)

(0.527)

(0.155)

Kilifi

-

0.276**

-

0.631***

0.048

-

0.454***

5.840***

-

0.122

-

0.153

0.404**

-

0.154

(0.083)

(0.102)

(0.176)

(0.147)

Constant

-

1.050***

-

3.624***

-

1.626***

(0.299)

(0.506)

(0.989)

(0.451)

𝜒

2

(36)

1834.52,

=

Prob >

𝜒

2

=

0.000

𝜒

2

(33)

62028.06,

=

Prob >

𝜒

2

=

0.000

𝜒

2

(30)

=

294.08,

Prob >

𝜒

2

=

0.000

Observations

276

334

136

-1.070**	-2.036***	-0.613**	-0.929**	-1.063**

(0.512)	(0.340)	(0.256)	(0.279)

Wald statistics

Notes: Other religion used as the base in the religion category; Baringo used as the base in the location fixed effects category (RVF and chikungunya) while Kwale is used as the base in the case of dengue fever in the location fixed effects category; Confidence intervals (95%) in parenthesis; * = significant at p < 0.1; ** = significant at p < 0.05; *** = significant at p < 0.00.
