# Supplementary material for: Evaluating livestock farmers knowledge, beliefs, and management of arboviral diseases in Kenya: A multivariate fractional probit approach
Source: PLoS Negl Trop Dis. 2021 Sep 16;15(9):e0009786. doi: 10.1371/journal.pntd.0009786 (PMC8478187; doi:10.1371/journal.pntd.0009786)
Supplement: Attachment — Submitted filename: Response to Reviewer Comments - PNTD - 21-00736.docx [file pntd.0009786.s003.docx]

**Response to Reviewer Comments – PNTD-D-21-00736**

**Evaluating Livestock Farmers Knowledge, Beliefs, and Management of Arboviral Diseases in Kenya: A Multivariate Fractional Probit Approach**

Dear Editor PNTD,

Thank you for giving us an opportunity to resubmit a revised version of the manuscript “*Evaluating Livestock Farmers Knowledge, Beliefs, and Management of Arboviral Diseases in Kenya: A Multivariate Fractional Probit Approach*” for consideration for publication in PLOS Neglected Tropical Diseases. We appreciate the time and effort that you and the reviewers dedicated to providing feedback on our manuscript and are grateful for the insightful comments and constructive suggestions, which help to improve the quality of this paper. In this letter, we respond to the specific reviewer comments (in a table format) and have also attached a revised version of the manuscript with tracked changes and a final revised clean copy. We hope that the changes we have made will improved the quality of the paper to the reviewer’s satisfaction and look forward to your publication decision.

|  | **Review Comment** | **Response** | **Line number** |
| --- | --- | --- | --- |
| **General comments** | | | |
| 1. | Sections 2.1 and 2.2: The authors should separate an equation with its supporting sentences from that of the next equation. This will make it easier to follow where one equation ends and the next one starts. | We have separated each equation’s supporting sentences from those of the next equation in the two sections as suggested. | Section 2.1 and 2.2 pages 5-8. |
| 2. | Page 9: It will be better to replace “sub-counts” with “towns”. If the authors want to retain “sub-counts”, then they should add further details about what they mean. | We have replaced the word “sub-counts” with “sub-counties”. These are the decentralized administrative units within a County in Kenya. | Section 2.3-page 9 |
| 3. | On section ‘2.3. Study Area and Sampling Procedure’, the authors should include an Ethics statement. The ethics statement should describe if there was informed consent from the study participants, and how the participants of the study consented to it or how the consent was obtained. It should also explain if there was ethical approval for the study, state the approving organization(s) and approval number (where applicable). | An informed consent was obtained from each responded before commencement of the interviews. We have included the ethical statement that includes the study protocol reference number in the last paragraph of section 2.3. | Section 2.3 pages 210-214. |
| 3. | The methodology was very well and in detail described. Potentially it could be made a little more concise as the manuscript overall is quite long, and about 1900 words of 5400 words in total are the methodology. | We have attempted to make the methodology section more concise by omitting three citations Kan and Fabrigar, 2017; Kassie *et al*., 2015 and Muriithi *et al*., 2018 that overemphasized points that had already been made | Section 2 |
| 4. | The authors presented the results starting with each Table. The approach is good and easy to follow. However, it seemed as if the table titles were repeated with each table number. To avoid this repetition and to make the Results sections easier to grasp, it will be better if the authors summarise the main findings of each table at the beginning of the paragraph instead. Then, they can put the Table number being referred to in brackets. Alternatively, the authors can maintain their format but replace the table headings with the summary of the findings of that table. | This comment from Reviewer 1 is acknowledged. We have made the changes as suggested by summarizing the main findings of each table at the beginning of each paragraph. | Section 3 Pages 12 (lines 266-268, 280-282) and 13 (lines 296-299) |
| 5. | In Figure 1, do the red spots on the three counties depict the sampled sites or sampled households? As if it were households these seem quite few on the county map, if they were 200 per county? It would additionally make sense to make the county maps bigger, for the sites to be more visible. | We acknowledge the comment by Reviewer 2. The red dots within the map of Kenya show the AD hotspot Counties (Baringo, Kilifi and Kwale). The pulled out maps show the sampled administrative sub-counties within the hotspot Counties. It was not possible to produce a study area map that fits all the 200 households in a sub-county. | Figure 1 |
| 6. | The authors were not generous with the interpretation of their Results. They mostly repeated the Results and sometimes, compared it to previous studies. Few suggestions on how to improve the Discussion section include:  It will be nicer if the authors started the Discussion with a summary of their results in the study in relation to the aim of their study.  The Discussion can be broken down into sub-sections that correspond with those in the Results, so that the particular Result being discussed can be easily identified in the article. Alternatively, since the focus of the study is intertwined amongst knowledge, belief and management of arboviral diseases, the authors can separate the Discussion into three groups to tackle the three areas well.  Summarize a particular finding, discuss what it implies, and then compare if it is consistent or in contrast with the results of other studies. See instances in the comments on page 17. | We thank reviewer 1 for this comment that suggests a great way of improving the presentation of our results. In the revised version of the paper, we have adopted the reviewers first recommendation where we begin the discussion with a summary of the results and then break them down into sub-sections that correspond to those specific results. We have cited the tables on which each of the discussed result can be seen. | Section 4 |
| 7. | The authors should indicate the limitation of their study before or after the recommendations. | Reviewers’ comment is well noted, and a revision containing the limitation of the study has been inserted at the last paragraph of discussion section. | Page 24 (lines 447-4455) |
| 8. | The authors discussed very well the studies public health relevance add how the data can be helpful to advance our understanding of the topic under study | We thank the reviewer for this compliment |  |
| **Editor’s comments** | | | |
| 1. | For future purposes, the authors should include line numbers in the manuscript to make it easier to review. | The editor’s comment is well noted. We have included line numbers throughout the document in the revised manuscript. | All pages |
| 2 | Kindly alternate the word “majority” with “many” or “most” to avoid redundancy | We have adopted the reviewers suggested style of alternating the words “majority” with “many” or “most” in improving the presentation of our results throughout the paper | Sections 4 and 5 |
| 3 | The word “significant” is often linked to the intensity of the results in the Results section. Outside the Results section, it will be better to replace it with similar words like “considerably”, “greatly”. | We have made revisions as follows. Replaced the word “significantly” in line 19 with “considerable”, replaced the word “significant” in line 28 with “great”, replaced the word “significant” in line 49 with “considerable”. However, as suggested by the editor the word “significant” has been retained in the results section. | Section 1 Pages 1-3 |
| 4 | The authors should provide full address of their affiliated institutions. For instance, in which town and country is icipe located? | We have included the town and country for the affiliated institutions in lines 5 and 6. | Page 1 |
| 5 | The email address of the corresponding author shows that he is affiliated to “icipe” but he did not include it as one of his affiliations. Correct accordingly. | We have revised this part by including “icipe” as part of the corresponding author’s affiliation. | Page 1 |
| 6 | The study was on livestock farmers according to the Conclusion section but this was not captured in the aim of the study. So, please rephrase the aim of the study to, “This study evaluated the drivers of livestock farmers’ KBM of ADs from a sample of 629 respondents selected using a three-stage sampling procedure in Kenya’s three hotspot counties of Baringo, Kwale, and Kilifi”. | We have inserted the words “livestock farmers” in the aim of the study as suggested by the editor and ensured that the words “livestock farmers” appear in all relevant sections. | Sections 1, 4 and 5 |
| 7 | All the listed keywords are already contained in the title. Kindly replace them with words that are not in the title but are within the abstract to make the manuscript more discoverable. Few suggestions include: public health, livestock farmers, awareness, Rift Valley fever, Chikungunya fever, Dengue fever. | This suggestion is well acknowledged. We have included “public health, livestock farmers, awareness, Rift Valley fever, Chikungunya fever, Dengue fever” as the key words. | Abstract, Page 1 |
| 8 | Page 4: The word, “present” in the last paragraph portrayed the preceding words as humans. Since the authors were trying to describe the content of the remaining sections of the manuscript, “contain” is a better word to use. (Please see the corrections in the MS Word version). | We have replaced the word “present” with “contain” as suggested. | Page 4 |
| 9 | Table 3: The authors should insert question mark (?) where necessary under the “characteristics” column. | Question marks have been inserted in Table 3 | Table 3 |
| **Reviewer 1** | | | |
| 1. | Unusual to call the disease caused by zika virus zika fever, especially as it only causes fever in less than 50% of cases. Maybe rephrase this sentence to use the arbovirus names rather than the disease they cause throughout sentence. | We have revised the sentence as recommended by using the arboviral names. | Page 2, lines 29-30 |
| 2. | Add location – globally? | We have added globally | Page 2, line 42 |
| 3. | Rephrase this sentence to clarify its meaning. | The sentence has been rephrased in the revised version of the paper | Page 2, lines 46-49 |
| 4. | Is this actually part of your results? Try to be consistent in using “we” or “third person” | This part has been deleted from the manuscript | Page 5 |
| 5. | It is expected that the following is explained in the method, result and discussion section, and thus this does not have to be described at the end of the introduction. I would delete this paragraph. | We have deleted the last paragraph of section one as recommended. | Section 1 |
| 6. | As manuscript is a little long, it may be worth shortening the method section a little, although it is explained very well, I would think about either putting it into | We have attempted to make the methodology section more concise by omitting three citations Kan and Fabrigar, 2017; Kassie *et al*., 2015 and Muriithi *et al*., 2018 that overemphasized points that had already been made | Section 2 |
| 7. | Maybe better mentioned in the limitation section of the discussion | This part has been moved to discussion section (last paragraph). | Section 4 |
| 8. | Is the survey attached to the manuscript? | The survey questionnaire is not attached but the data used was provided |  |
| 9. | Please further explain the qualification for the health promotion, as this may be a biased proxy for social capital and networking? | Health promotion group constituted individuals who received training from health specialists, thus increasing awareness of good health, diet and exercise in the society. | Page 11, lines 246-248 |
| 11. | Restructure throughout MS: The socioeconomic characteristics of X households in the three Ads hotspots in Kenya displayed that overall 55 percent of the households were aware of the health impacts of Ads while 33 percent of the households had suffered from at least one of them (Table 1).  For X add number of households. | Restructured and added the number of households (629) as recommended by the reviewer. | Page 12, lines 253-255. |
| 12 | Restructure like explained above | Restructured as advised by reviewer 1 | Page 12, lines 280-282 |
| 13 | Change this nomenclature as described above | This comment is well taken, and the nomenclature has been revised as advised | Section 4 |
| 14 | of | Replaced “in” with “of” | Page 12, line 288 |
| 15 | Restructure – “The study of farmer’s specific knowledge of signs, symptoms, transmission methods, beliefs and management practices of the three ADs in Kenya showed that the main sign of the three ADs in human were fever, abdominal pains and headache as reported by 82, 29 and 19 percent of the respondents, respectively (Table 3).” | This comment is well taken, and the suggestion has been included in the revised document | Page 13, lines 296-299 |
| 16 | Explain, what is bush clearing? | We have explained bush clearing as the cutting of overgrown vegetation around the homestead that can act as hiding ground for mosquitoes | Lines 314-315 |
| 17 | Spell out what you are trying to say in this sentence. | We have included the relevant citation | Page 21, Line 374-375 |
| 18 | Why? Are the mosquitos day active or not used correctly? | We have revised this by adding that most ADs mosquitoes feed during the day | Page 21, line 384 |
| 19 | In which direction? | Positively or negatively | Page 24, line 443 |
| 20 | You have to make the link in your argumentation, If health seeking | This comment is well taken, and the link in the argument has been made as advised | Section 5 |
| **Reviewer 2** | | | |
| 1. | Delete “(MVFP)” since the abbreviation was not used anywhere else in the abstract. | Deleted | Page 1, line 16 |
| 2. | Add citations for these sentences | The citations have been added as recommended | Page 2, lines 45 and 46 |
| 3. | Rephrase to “…an estimated 40,000 deaths due to dengue …” | We acknowledge this comment and rephrase accordingly | Page 2, line 346 |
| 4. | The authors wrote more than one effect. So, they should correct the phrase, “…zoonotic effect of arboviral diseases (ADs) includes…” to “…zoonotic effects of arboviral diseases (ADs) include…” | We have removed letter “s’’ from include | Page 2, line 46 |
| 5. | This is not in the list of references. Either include in the reference list or replace with a more suitable reference. | This reference (FAO, 2016) has been added | Reference section |
| 6. | The authors should kindly specify the name of the model that they meant. | We have deleted “above” and inserted “MVP” | Page 7, line 157 |
| 7. | Explain the meaning of this word | Thank you for this comment, we have deleted the word” aew” and inserted “are” | Page 8, line 175 |
| 8. | Kindly replace with “town” or provide more details of “sub-counts” meaning. | We have provided more details about the meaning of sub-counties. These are decentralized administrative units within a County in Kenya. | Page 9, line 201. |
| 9. | Cite this software and include the corresponding reference. | Citation provided | Page 10, line 208 |
| 10. | Indicate the percent in brackets. | The percentage has been included | Page 15, line 306. |
| 11. | Kindly put the P-value in brackets so that the audience will know what you mean in subsequent appearances. | We have included the P-value as recommended by Reviewer 2. | Page 18, line 339. |
| 12. | “Moreover” cannot be used to start a paragraph because it is comparing the sentence it is used in with a supposed previous sentence or statement. So, please delete. | Deleted as advised | Page 20, line 348. |
| 13. | Cite these earlier studies. | We have provided the appropriate citation | Page 20, line 367, 368. |
| 14. | This is not the Results section. It will be better to replace this value with “most”. | Deleted and replaced as recommended | Page 21, line 371. |
| 15. | The authors should replace this phrase with a summary of how the reports of these 2 cited studies were consistent with their own result and the 2 sentences before it. | We have rephrased this part by providing addition information before citing these studies. | Page 21, line 377. |
| 16. | Kindly rephrase to:  For instance, household education level increased the intensity of knowledge, beliefs and management of Ads, which was consistent with previous studies (Higuera-Mendieta et al., 2016; Kumaran et al., 2018; Nguyen et al., 2019). | Rephrased as advised | Page 22, line 407-408. |
| 17. | Using “between” means that’s the 2 numbers are not included. If you intend to include the two stated numbers, kindly replace with “from 31 to 154 percent”. | Replaced accordingly in the results section | Page 22 |
| 18. | Expand on this statement by indicating what the other studies found. For instance, modify the statement to “This was consistent with the reports of Abdi et al., 2015 and Harapan et al., 2018 that reported that …” | We acknowledge this comment and modified the statement as recommended by Reviewer 2. | Page 23, lines 421-423 |
| 19. | This paragraph was well written. The authors can adapt this format in the other paragraphs of the Discussion. | Comment is well received. Thanks |  |
| 20. | Replace with word because it is one digit and you wrote the unit in words instead of using the symbol. | Replaced accordingly | Page 23, line 424 |
| 21. | Either write two percent or 2%. | Rewritten as two percent | Page 22, line 410 |
| 22. | See comments above | Rewritten as three percent | Page 22, lines 410 |
| 23. | Add a sentence afterwards on what this ‘negative relationship’ means. | We acknowledge this comment and have added a statement that explains the negative relationship. The finding This suggested that as distance increased, the likelihood of the household members visiting health facilities declined and thus less likely to manage the diseases | Page 23, lines 433-435 |
| 24. | As it is, these statements fit better in the Discussion. For Conclusion section, kindly merge them and modify to:  “We demonstrated the importance of access to information (experience, awareness and distance to a health facility), income, education, and religion in influencing the KBM of ADs”. | We acknowledge this comment and revised accordingly | Page 24 lines 468-469. |

.
